# Evolution of bispecific and multispecific antibodies in cancer therapy

**DOI:** 10.1016/j.lanepe.2026.101599

**Published:** 2026-03-19

**Authors:** Elisa Fontana, Rafael Grochot, Nuria Kotecki, Bernard Doger, Simon Nannini, María de Miguel

**Affiliations:** a Sarah Cannon Research Institute (SCRI), London, United Kingdom; b Institute Jules Bordet, Brussels, Belgium; c START Madrid-Fundación Jiménez Díaz, Hospital Universitario Fundación Jimenez Diaz, Madrid, Spain; d START Rioja, Centro de Investigación Biomédica de La Rioja/Hospital San Pedro, Logroño, Spain

**Keywords:** Antibody, Bispecific, Multispecific, Antigen, CD3, Phase I Clinical Trial, Clinical Trial Phase II, Clinical Trial Phase III, Drug development, Oncology, Target

## Abstract

Antibody-based cancer therapy has rapidly evolved from monoclonal antibodies to bispecific and multispecific constructs that combine distinct binding specificities and mechanisms. These agents are seeing increasing clinical adoption, with European Medicines Agency approvals in haematological malignancies and selected solid tumours such as uveal melanoma and EGFR-mutant non-small-cell lung cancer. However, they are often still discussed as a single drug class, which does not capture the complexity of current formats and mechanisms, ranging from IgG-like to fragment-based architectures and from immune-cell redirection to dual immune modulation or oncogenic pathway blockade. This Series paper provides an integrated classification framework based on mechanism and format, relating key design features to pharmacology, efficacy, and safety. It synthesizes clinical evidence and ongoing development, discusses practical strategies to mitigate hallmark toxicities, and reviews emerging resistance mechanisms and rational combination approaches. It also outlines next generation directions, including higher order multispecific constructs, conditionally active antibodies, and payload conjugated multispecific formats. To consolidate these agents as an established therapeutic modality in oncology, priority should be given to rigorous understanding of mechanisms of action and toxicity, alongside rational optimisation of construct design and dosing, supported by robust prospective translational programmes.

## Introduction

The introduction of antibody-based therapies has redefined cancer therapeutics by enabling selective modulation of key oncogenic pathways and improved immune responses. Among these, bispecific antibodies (bsAbs), represent a significant step forward, designed to engage two distinct molecular targets or epitopes simultaneously. Dual targeting capability has opened new possibilities to improve efficacy by promoting synergistic pathway inhibition, overcoming resistance mechanisms, and activating immune effector cells. Despite the clinical success of first-generation bsAbs across hematologic and solid tumors, challenges such as antigen heterogeneity, adaptive resistance, and systemic toxicity remain significant barriers. To overcome these limitations, next generation multispecific antibodies (msAbs) are being developed with enhanced valency, modular architectures, and spatially controlled activation strategies. This article explores the evolution of bsAbs and msAbs, highlighting emerging engineering strategies that may unlock their full future potential.

## Classifications of bispecific and multispecific antibodies

### Structural classification

BsAb and msAb are categorized by specificity, valency, affinity, and structurally by whether they retain an IgG-

**Key messages**

• The functional diversification of bsAbs and msAbs enables simultaneous immune activation, tumor microenvironment modulation, or dual oncogenic blockade, to achieve better clinical outcomes.
• Several bsAbs are already approved and have shown meaningful clinical efficacy across a broad spectrum of tumors. Early-phase trials are exploring novel bsAbs that aim to enhance immunomodulation, increase affinity for current or emerging TAAs, or broaden immune cell engagement.
• Safety is a key factor for antibody therapy development, with CRS, on-target/off-tumor toxicity, IRR and immunogenicity mitigated through rational antibody engineering (e.g., Fc silencing, asymmetric formats), optimized affinity, and tailored dosing strategies such as step-up, split doses, or subcutaneous administration to balance drug exposure and tolerability.
• Resistance mechanisms include tumor-intrinsic factors (antigen loss, MHC-I downregulation), immune dysfunction (T-cell exhaustion), and drug clearance (sink effect, FcγR-mediated degradation), highlighting the need for combination strategies and refined designs to improve clinical outcomes.
• Innovative strategies include construct scaffolding—such as the adjunction of a payload, and masking designs to mitigate the risk of toxicity—as well as combination therapies aiming for tumor burden reduction, target antigen upregulation, or synergistic activity.

**Search strategy and selection criteria**

A comprehensive literature search for this Series article was performed up to May 1st 2025 though PubMed/MEDLINE library with the search terms “bispecific”, “bispecific antibodies”, or “multispecific”, alone or adding one of the following: “mechanism of action”, “class”, “structure”, “cell engagers”, “NK cell”, “dual”, “receptor-ligand”, “tumor targeting”, “clinical development”, “mechanism of resistance”, “safety”, “Toxicity”, “next generation”, “combination”. Adding filters: “Review”, “Clinical Trial—Phase I”, “Clinical Trial—Phase II”, “Clinical Trial—Phase III”. The search yielded 464 results when limited by date (from 2020). Additional searches were performed allowing for previously dated articles for specific sections. Abstracts and proceedings from major oncology conferences [American Society of Clinical Oncology (ASCO) Annual Meeting, European Society of Medical Oncology (ESMO)] Congress were also screened. FDA and EMA European public assessments reports (EPAR) of EMA-authorized bispecific antibodies for solid tumors and hematological malignancies. Only articles published in English and containing an abstract were included. All studies were screened by the six authors and were only excluded if they were found to be beyond the intended scope. Because only a minority of bsAbs currently have mature, randomised phase 3 data, many of the clinical outcomes reported here derive from early-phase, often single-arm studies and should be regarded as illustrative and hypothesis-generating rather than as a basis for formal comparative efficacy inferences.

like architecture or adopt engineered non-IgG formats, each with distinct pharmacological and clinical properties.

IgG-like formats maintain the overall structure of a full-length IgG, including the Fc region, which confers prolonged half-life and effector functions like antibody-dependent cell-mediated cytotoxicity (ADCC) and complement-dependent cytotoxicity (CDC). These formats can be further subclassified into symmetric and asymmetric architectures. Symmetric IgG-like bsAbs resemble natural antibodies and are typically easier to produce with high expression yields, though their close spatial proximity of binding sites can sometimes compromise bispecificity and functional potency. In contrast, asymmetric formats address challenges of heavy and light chain mispairing through sophisticated engineering strategies like Knobs-into-Holes, CrossMab, or DuoBody technologies, enabling optimized binding to distinct targets, but more complex to produce.^1^^,^^2^ These structural differences directly affect clinical efficacy, toxicity, and the need for specific dosing or preconditioning strategies. Asymmetric formats, such as the 2:1 CD20 × CD3 design of glofitamab, allow bivalent binding to the tumor antigen and monovalent CD3 engagement, increasing tumor selectivity and potency but also raising the risk of cytokine release syndrome (CRS). This requires pre-treatment with obinutuzumab to deplete peripheral B cells and reduce CRS risk. In contrast, mosunetuzumab uses a symmetric 1:1 format with reduced CD3 affinity, leading to a more favourable safety profile.^3^ Non-IgG-like bsAbs lack the Fc region and are built on compact scaffolds such as single-chain variable fragments (scFvs), resulting in smaller molecular size, enhanced tissue penetration, and short plasma half-lives. Examples are bispecific T cell engagers (BiTE), dual affinity retargeting antibodies (DART), bi-Nanobodies, and Tandem diabodies (TandAb).^4^

MsAbs are designed to engage three or more targets simultaneously to enhance therapeutic efficacy. Trispecific and tetraspecific antibodies have entered clinical research, while advanced modular multispecific platforms enable the flexible assembly of custom-designed building blocks to generate msAbs.^5^

### Mechanistic classification

**Fig. 1:**
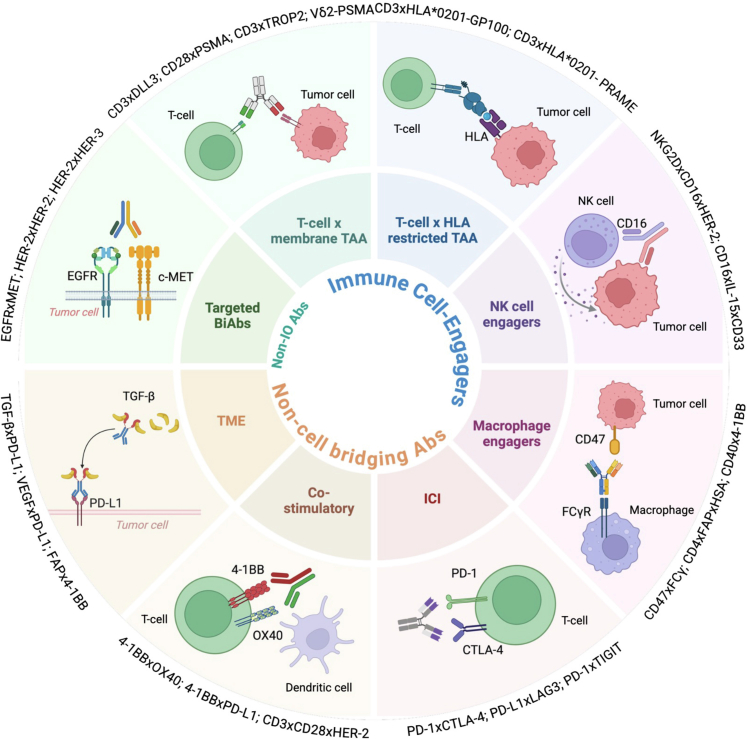
**Classification of bsAb and multispecific antibodies by mechanism of action.** Bispecific (bsAbs) and multispecific antibodies (msAbs) are organized into three main functional categories: immune cell engagers, non-cell bridging antibodies, and non-immune-oncology (non-IO) antibodies. Immune cell engagers include T-cell engagers directed against membrane-bound tumor-associated antigens (TAA) or HLA-restricted TAAs, natural killer (NK) cell engagers, and macrophage engagers. Non–cell bridging antibodies encompass co-stimulatory agonists, immune checkpoint inhibitors (ICIs), and tumor microenvironment (TME) modulators, which do not necessarily establish direct physical linkage between immune effector cells and tumor cells. Non-IO antibodies primarily comprise targeted therapies that block oncogenic signaling pathways, such as EGFR, MET, or HER family receptors. Selected molecular targets and examples of bsAbs and msAbs under development are indicated within each sector.

BsAbs and msAbs can be broadly categorized according to whether their primary action involves direct immune cell recruitment to tumor cells, modulation of immune signaling pathways, or direct blockade of oncogenic receptor–ligand interactions (Fig. 1).

#### Cell-engaging therapies

T-cell engagers (TCE) aim to physically facilitate the formation of the immune synapses between immune effector cells and tumor cells, promoting direct cytotoxic activity.^6^ Full T-cell activation requires both signal 1, provided by an antigen-presenting cells (APCs) to the T-cell receptor (TCR) and signal 2, via co-stimulatory signaling such as CD28 or 4-1BB receptors.^7^ The first and most clinically advanced cell engagers primarily focused on T-cell activation via binding to the CD3 subunit of the TCR and relying on the tumour cells to provide signal 2. A different class of TCEs bridges a costimulatory receptor on the T-cell and a cancer antigen on the cancer cell, providing a costimulatory signal via signal 2 and facilitating signal 1 via the proximity of the cancer cell.

A substantial proportion of bsAbs target membrane-associated proteins as tumor-associated antigens (TAAs), although these antigens represent only 10% of the proteome considered amenable to therapeutic targeting.^6^ To expand the antigenic landscape, alternative cell-engaging strategies such as immune mobilizing monoclonal T-cell receptors (TCR) against cancer (ImmTACs) have been developed, enabling the recognition of intracellularly derived peptides presented in the context of HLA,^7^ most frequently HLA-A∗02:01. ImmTACs are engineered high-affinity TCR fused to anti-CD3 domains that redirect polyclonal T cells to peptide-HLA complexes, as exemplified by tebentafusp, a CD3 x HLA ∗02:01 GP100, which improved survival in metastatic uveal melanoma. However, their use is limited to patients expressing the matching HLA allele, thus narrowing the eligible population.^8^ Finally, other emerging msAbs binding CD3 and two different TAAs are being engineered to enhance tumor selectivity for cancer cells over normal cells due to co-expression of both TAAs, hence reducing on-target/off-tumor toxicity.^9^

Natural Killer cell engagers (NKCE) exploit the innate cytotoxic potential of NK cells by bridging them to tumor cells. NKCEs typically target activating receptors on NK cells as CD16a (FcγRIIIa) or NKG2D predominantly, but also NKp30, and NKp46, each offering distinct activation strengths and signaling pathways.^10^ CD16a is the most extensively utilized receptor, mediating strong activation but subject to rapid downregulation; NKG2D and natural cytotoxicity receptors like NKp30 and NKp46 offer alternative activation strategies with potentially improved persistence and recruitment of additional immune subsets.^11^ A variety of NKCE formats, including BiKEs (bispecific killer engagers) and TriKEs, are under development. While BiKEs typically follow a NK receptor x TAA format, TriKEs incorporate a binding domain for the TAA and either co-engage two NK cell receptors (e.g., NKG2D and CD16) or include an IL-15 moiety to promote NK cell proliferation and persistence.^5^^,^^12^

Macrophage-engagers are designed to recognize, engulf, and destroy tumor cells by blocking inhibitory signals such as CD47 and by directly triggering phagocytosis via Fcγ receptors or activating receptors. Targeting and blocking the interaction between CD47 on tumor cells and SIRPα on macrophages, can effectively abrogate the “don't eat me” signal, restoring macrophage-mediated phagocytosis. Secondly, macrophage engagement through Fcγ receptors (FcγRs), particularly FcγRI-III, triggers antibody-dependent cellular phagocytosis (ADCP) upon antibody Fc domain binding, thereby promoting tumor cell internalization and destruction.^13^ Due to the high expression of CD47 on normal cells (especially endothelial cells, red blood cells and platelets) a number of monoclonal antibodies targeting CD47 failed to demonstrate significant clinical activity with high on-target/off-tumour toxicity such as anemia and thrombocytopenia. By using a bispecific antibody with high affinity for a TAA and low affinity for CD47, bispecific macrophage-engagers are restoring optimism in this field. Additionally, emerging strategies focus on the direct stimulation of macrophage-activating receptors such as CD40, MerTK, or C-type lectin-like receptors, reprogramming macrophages toward a pro-inflammatory and tumoricidal phenotype within the tumor microenvironment.^13^^,^^14^

#### Non-cell-bridging therapies

Academically, it can be subdivided into immune checkpoint inhibitors (ICIs), co-stimulatory agonists, or tumor microenvironment (TME)-targeting bsAbs/msAbs. However, combinations of these mechanisms are frequently employed within a single molecule to enhance immune responses through multiple complementary pathways. Targeting two (e.g., PD-1 x CTLA-4) or even three ICI formats (e.g., PD-1 x CTLA-4 x TIGIT) can generate a greater synergistic immune activation and help overcome resistance mechanisms associated with checkpoint upregulation of alternative inhibitory pathways (e.g., PD-L1 x LAG-3, PD-1 x TIGIT, or PD-L1 x TIM-3).^15^^,^^16^

BsAb agonists targeting co-stimulatory receptors represent a promising but challenging approach to amplify anti-tumor immunity by delivering signal 2, the co-stimulatory signal required for full T-cell activation (e.g., OX40 x 4-1BB).^17^ Nevertheless, the clinical translation of dual co-stimulatory agonists remains limited, due to their potential for uncontrolled systemic immune activation, as in the dramatic episode of TGN1412, where the drug acted as CD28 superagonist, leading to polyclonal and unspecific immune activation in absence of signal 1.^17^^,^^18^ As a result, other target combinations are being explored to improve safety, including TAA-restricted co-stimulatory agonists (e.g., TAA x CD28, TAA x 4-1BB), combinations of ICI and co-stimulatory agents (e.g., PD-L1 x 4-1BB), combinations of TAA x CD3 and TAA x CD28, and integrated trispecific constructs that simultaneously engage CD3 and CD28 for dual T-cell activation while targeting the TAA.^19^^,^^20^ These approaches aim to provide both signal 1 and signal 2 in a spatially and temporally controlled manner. Preclinical studies support this dual-targeting strategy to improve efficacy and mitigate T-cell exhaustion.^21^

TME-targeting bsAbs aim to counteract the immunosuppressive microenvironment, which is a key resistance driver. To overcome these barriers, bsAbs and msAbs are under development, such as PD-L1 × VEGF (ivonescimab), TGF-β x PD-L1 (bintrafusp-alfa), and novel agents addressing fibroblast activating protein (FAP x 4-1BB, FAP x CD40).^22^

#### Receptor-ligand blockade

Targeted bsAbs simultaneously inhibit different receptor–ligand interactions critical for tumor growth or survival (e.g., EGFR x MET). They may block redundant or compensatory pathways to overcome resistance mechanisms, thereby improving clinical outcomes. Biparatopic antibodies target two different epitopes on the same receptor (e.g., HER2 × HER2, MET x MET) to induce more effective receptor crosslinking, internalization, and degradation.^23^ Other agents prevent heterodimerization, critical for downstream oncogenic signaling (e.g., HER2 x HER3).^24^ Finally, bsAbs can bind simultaneously to a receptor and its ligand, trapping them in an inactive complex; this is exemplified by the VEGF x Angiopoietin-2 bispecific (faricimab), which is currently approved for indications outside oncology.^25^

## Recent achievements in clinical development

**Fig. 2:**
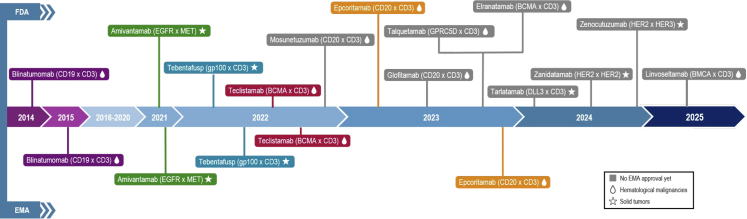
**Timeline of approvals for multispecific antibodies over the last 10 years, by both the U.S. Food and Drug Administration (FDA) and the European Medicines Agency (EMA)**.

Proximity of target antigens is crucial to allow for the originally rigid structure of a bsAb to engage two distinctive cells. This is possibly the reason for which hematological malignances, by having cancer cells close proximity of lymphocytes, to be particularly suitable for bsAbs’ MoA. Blinatumomab, a B-cell and T-cell engaging antibody (CD19 x CD3) was the first approved bsAb, for patients with Philadelphia chromosome (Ph)-negative B-cell precursor acute lymphoblastic leukemia (ALL) (Fig. 2).^26^ The approval was subsequently granted to Ph-positive ALL. Seven years later, teclistamab was approved for the treatment of relapsed/refractory (R/R) multiple myeloma, followed by elranatamab, both targeting the B-cell maturation antigen (BCMA) on myeloma cells and CD3 on T-cells. While indirect comparisons suggest that elranatamab may offer advantages in terms of response rates and survival outcomes, these findings should be interpreted cautiously due to the absence of head-to-head comparative trials and the confounding impact of the COVID-19 pandemic on the efficacy outcomes reported for teclistamab. The approval for talquetamab (GPRC5D x CD3) followed these two bsAbs for the same indication.^27^ Three bsAb targeting the B-cell antigen CD20 have been approved for R/R lymphomas: mosunetuzumab, for follicular lymphoma, glofitamab and epcoritamab, for diffuse large B-cell lymphoma.

To successfully move into the solid tumor space, cell-bridging bsAbs required highly immune-infiltrated tumors. Not surprisingly, the first approvals for immune cell engagers were in melanoma and small cell lung cancer (SCLC). In a pivotal phase 3 trial, tebentafusp significantly improved outcomes in metastatic uveal melanoma, with 37% increase in progression-free survival (PFS) rate and 49% increase in overall survival (OS) rate compared to investigator's choice (immune checkpoint inhibitor or dacarbazine).^7^ Tarlatamab, a DLL3 × CD3 T-cell engager, received accelerated approval from the FDA for R/R SCLC following platinum-based therapy. The phase 3 DeLLphi-304 trial demonstrated a significant improvement in OS with tarlatamab compared to chemotherapy in the second-line treatment of patients with R/R SCLC. The observed absolute gain in median OS of 5.3 months, along with a 40% reduction in the risk of death, mark a breakthrough in a disease historically lacking durable responses.^28^

The rational for clinical success of non-cell bridging bsAbs in solid tumors was based on a number of long-established monoclonal antibodies. Amivantamab, an EGFR x MET bsAb with dual signaling inhibition and ADCC activity, is the first FDA and European Medicines Agency (EMA) approved bsAb for a solid malignancy: the phase 1 CHRYSALIS trial demonstrated an objective response rate (ORR) of 40%, a median duration of response (mDoR) of 11.1 months, and a mPFS of 8.3 months in non-small cell lung cancer (NSCLC) harboring an *EGFR* exon 20 insertion. Its role has since expanded through three phase 3 studies: PAPILLON (in combination with platinum-based chemotherapy), MARIPOSA (in addition to lazertinib for exon 19 del/L858R NSCLC), and MARIPOSA-2 (combined with platinum doublet in the post-osimertinib setting).^29^^,^^30^

A high number of clinical trials are ongoing (Table 1), including second-generation bsAbs. MCLA-129 (EGFR x cMet), for example, has increased potency and enhanced ADCC activity compared to amivantamab.^31^ A phase 1/2 clinical trial is ongoing in patients with solid tumors harboring *EGFR* or *cMet* mutations, for which durable antitumor activity has been observed.^32^ Bafisontamab (EMB-01, EGFR x cMet) has shown preliminary antitumor activity by targeting both *EGFR* wild-type/mutant isoforms and inducing co-degradation of both EGFR and cMet receptors.^33^

**Table 1:** General overview of active early phases clinical trials in solid tumors investigating bispecific or multispecific antibodies.

Targets	MoA	Study drug (NCT number)
**Cell engaging strategies**		
PSMAxCD3	T cell engager	REGN4336 (NCT05125016); CC-1 (NCT04104607/NCT05646550)
PSMAxCD28	T cell engager	REGN5678 (NCT05125016)
PSMAxVγδ	γδ T cell engager	LAVA-1207 (NCT05369000)
CLDN6xCD3	T cell engager	CTIM-76 (NCT06515613); XmAb541 (NCT06276491)
CLD18.2xCD3	T cell engager	AZD5863 (NCT06005493)
EGFRxCD3	T cell engager	EGFR FPBMC (NCT06479239)
EGFRxCD3	T cell engager/BATs	EGFR BATs (NCT03269526)
EGFRxCD28	T cell engager	REGN7075 (NCT04626635)
EGFRxVδ2	T cell engager	PF08046052 (NCT05983133)
CDH17xCD3	T cell engager	Cabotamig (NCT05411133)
CEAxCD3	T cell engager	BA1202 (NCT05909241); NILK-2301 (NCT06663839)
ENPP3xCD3	T cell engager	JNJ-87890387 (NCT06178614); XmAb819 (NCT05433142)
EpCAMxCD3	T cell engager	BA3182 (NCT05808634); M701 (NCT06266091/NCT06432296)
FAPxCD3	T cell engager	NG-641 (NCT05043714)
MUC16xCD3	T cell engager	Ubamatamab (NCT03564340)
PDL1xCD3	T cell engager	BS-006 (NCT05393440)
B7H3xCD3	T cell engager	CC-3 (NCT05999396)
B7H3xCD28	T cell engager	XmAb808 (NCT05585034)
B7H4xCD3	T cell engager	GEN1047 (NCT05180474)
CLDN6xCD137xCD3	T cell engager	SAIL66 (NCT05735366)
cMETxCD16axNKG2D	TriNKET	ABBV-303 (NCT06158958)
HER2xCD16xNKG2D	TriNKET	DF-1001 (NCT04143711)
**Checkpoint inhibition and immunomodulatory strategies**		
**Dual checkpoint inhibition**		
PD-1xCTLA4	Adaptive	Cardunilizumab (NCT06114173); Lorigelimab (NCT05293496); SI–B003 (NCT04606472); Volrustomig (NCT06535607); QL1706 (NCT06786026)
PD-1xTIM-3	Adaptive	LB1410 (NCT06468358); Lomvastomig (NCT03708328); Sabestomig (NCT04931654/NCT04931654)
PD-1xLAG3	Adaptive	INCA32459-101 (NCT05577182); Tobemstomig (NCT04785820); RO7247669 (NCT04140500); Tebotelimab (NCT040823643); AK129 (NCT05645276)
PD-1xTIGIT	Adaptive	Rilvegostomig (NCT04995523/NCT05123482)
PD-1xCD47	Adaptive/Innate	HX009 (NCT05731752)
PD-1xILT4	Adaptive/Innate	CDX-585 (NCT05788484)
PD-L1xILT4	Adaptive/Innate	SPX-303 (NCT06259552)
PD-L1xCD47	Adaptive/Innate	BAT7104 (NCT05767060); IMM2520 (NCT05780307); IMM2902 (NCT05805956)
PD-L1x4-1BB	Adaptive	ABL503 (NCT04762641); Acasunlimab (NCT05117242); PRS-344 (NCT05159388)
PD-L1xOX40	Adaptive (agonist)	EMB-09 (NCT05263180)
B7-H4x4-1BB	Adaptive	ABL103 (NCT06126666)
ILT2xILT4	Innate	PF-07826390 (NCT06546553)
**Co-stimulatory molecules**		
PD-L1xCD137	Adaptive	FS120 (NCT04648202); FS222 (NCT04740424)
PD-1xVEGF	Adaptive	AI 081 (NCT06635785); JS207 (NCT06022250); LM-299 (NCT06650566); AP505 (NCT06723964)
PD-L1xVEGF	Adaptive	AP505 (NCT06723964); PM8002 (NCT05918445; NCT06712316), IMM2510 (NCT05972460)
PD-1xCTLA4xVEGF	Adaptive	HC010 (NCT06307925)
PD-L1xTGFb	Adaptive	Y101D (NCT05028556)
PD-1xTGFbxVEGF	Adaptive	DR30106 (NCT06132828)
PD-L1xIL2	Adaptive	IBI363 (NCT06081907/NCT06468098/NCT06797297/NCT06717880/NCT05290597)
**Target inhibition**		
PD-1xHER2	Adaptive	SSGJ-705 (NCT06390774)
4-1BBxEGFR	Adaptive	BNA035 (NCT05150457)
4-1BBx5T4	Adaptive	ALG.APV-527 (NCT05934539)
4-1BBxHER2	Adaptive	YH32367 (NCT05523947)
MesothelinxCD47	Innate	NI-1801 (NCT05403554)
DLL3/CD47	Innate	Peluntamig (NCT05652686)
CLDN18.2XCD47	Innate	Spevatamig (NCT05482893)
**Signal pathway inhibition**		
EGFRxcMET	Dual receptor inhibition	[225Ac]-FPI-2068 (NCT06147037); EMB-01 (NCT05176665/NCT03797391); HS-20117 (NCT06621563); LY3164530 (NCT02221882; MCLA-129 (NCT04868877/NCT04930432)
EGFRxHER3	Dual receptor inhibition	IBI3005 (NCT04603287); SI-B001 (NCT04603287)
EGFRxLGR5	Dual receptor inhibition	MCLA-158 (NCT03526835)
HER2xHER3	Dual receptor inhibition	MCLA-128 (NCT02912949)
EGFRxcMETxcMET	Biparatopic BsAb	BG-T187 (NCT06598800); GB263T (NCT05332574)
HER2xHER2	Biparatopic BsAb	KM257; MBS301 (NCT03842085); TQB3616 (NCT06202261); ZW49 (NCT03821233); KN026 (NCT03847168)
EGFRxcMET	ADC	GEN1286 (NCT06685068)
HER2xTrop2	ADC	JSKN016 (NCT06592417)

Abbreviations: MoA, Mechanism of Action; BATs, Bispecific antibody Armed T-cells; TriNKET, Tri-specific NK Engager Therapy; ADC, antibody-drug conjugate.

Zanidatamab, a biparatopic HER2-targeting bsAb that binds two distinct extracellular HER2 epitopes, received FDA approval for pretreated HER2-positive biliary tract cancer (BTC). In the HERIZON-BTC-01 trial, an ORR of 41% and median PFS of 5.5 months were demonstrated.^34^ Two ongoing phase 3 trials (HERIZON-BTC-302 and HERIZON-GEA-01) are evaluating its efficacy in combination with chemotherapy for first-line HER2+ BTC and gastroesophageal adenocarcinomas (GEA), respectively.^35^^,^^36^ Zenocutuzumab-zbco, a HER2 × HER3 bsAb, was subsequently FDA-approved for *NRG1* fusion-positive pancreatic cancer and NSCLC. In the eNRGy trial, zenocutuzumab showed ORRs of 40% and 33%, and mPFS of 6.8 and 9.2 months, respectively.^24^

Cadonilimab (AK104), a tetravalent PD-1 × CTLA-4 bsAb, was approved by the Chinese National Medical Products Administration (NMPA) for advanced gastric/gastroesophageal junction (G/GOJ) adenocarcinoma and metastatic cervical cancer; FDA and EMA approvals have not yet been announced. In G/GOJ cancer, cadonilimab combined with chemotherapy improved OS by 34% while reducing immune-related adverse events compared to the traditional dual checkpoint blockade.^37^ In cervical cancer, cadonilimab showed efficacy both as monotherapy in pretreated patients and in combination with chemotherapy in the first-line setting, supporting its dual regulatory approvals.^38^

Most recently, ivonescimab, a PD-1 × VEGF bsAb designed to overcome immunoresistance mediated by angiogenesis, received NMPA approval for previously treated *EGFR*-mutant NSCLC. In the HARMONi-A trial, ivonescimab combined with chemotherapy improved PFS by 54% and ORR by 15% compared to chemotherapy alone.^39^ A priority review is underway for first-line PD-L1-positive advanced NSCLC, based on interim results from the HARMONi-2 trial, showing a 49% improvement in PFS versus pembrolizumab.^40^

In hematology, next-generation CD3 bsAbs with enhanced tumor-associated antigen specificity are also advancing in late-stage development. In lymphoma, the phase 3 OLYMPIA-1 and OLYMPIA-3 trials are evaluating odronextamab (CD20 × CD3) versus standard regimens in follicular lymphoma and DLBCL, respectively^41^; indirect comparisons have shown higher complete response rates than mosunetuzumab and epcoritamab. In multiple myeloma, linvoseltamab (BCMA × CD3) demonstrated promising antitumor activity in early trials and is now under phase 3 evaluation (LINKER-MM3) against elotuzumab.^42^ It has recently received FDA accelerated approval and EMA conditional authorisation based on the LINKER-MM1 and MM2 studies.

## Challenges and considerations

### Safety and toxicity

Despite their growing clinical relevance, bsAbs are associated with a complex spectrum of toxicities that require careful consideration during drug development and clinical implementation. CRS is a supraphysiologic immune response characterized by excessive and rapid activation of T-cells and other effector cells, leading to hypersecretion of proinflammatory cytokines such as IL-6, IL-10, IL-18, IFN-γ, and TNF-α. Clinically, CRS often begins with fever and can escalate rapidly to hypotension, disseminated intravascular coagulation, and acute respiratory distress syndrome with hypoxemia requiring mechanical ventilation.^43^ Comprehensive drug design and thorough preclinical evaluation are essential prior to clinical testing, minimizing unnecessary toxicity.

Optimizing the balance between tumor antigen and immune effector affinity is critical. While high affinity bsAbs (e.g., CD3 × tumor antigen) enhance immune activation, they also increase CRS risk. On the contrary, lower-affinity CD3 binders maintain efficacy in antigen-rich tumors with reduced toxicity, if the antigen binding remains sufficient for effective engagement.^44^ An FDA analysis of 17 different CD3-targeting bispecific investigational products in oncology examined a number of product characteristics, in-vitro, in-vivo and clinical data in relation to risk of CRS.^45^ Modifiable antibody characteristics included valency, incorporation of an interleukin-trapping domain to reduce systemic IL-6 exposure, and relative binding affinity for CD3 compared to the TAA, with better tolerability when the affinity for CD3 is lower than that one for the TAA. Examples include a novel TROP2 × CD3 bsAb and a PSMA × CD3 bsAb in preclinical development, both engineered with attenuated CD3 affinity, as well as a CD276 × CD3 bsAb currently in phase I evaluation (NCT05999396),^46^ designed with a 100-fold reduced CD3 binding. Moreover, binding different epitopes within the CD3 protein complex can lead to distinct affinity ranges: targeting the CD3ε domain with reduced affinity is a strategy to uncouple T-cell killing from cytokines release.^47^ ISB-1342, a CD38 x CD3 bsAb currently in development for R/R multiple myeloma (MM), carries a detuned scFv domain affinity binding to CD3ε on T-cells. Preliminary results from dose-escalation revealed a 34% incidence of CRS, all being grade 1–2 with no grade 3 or higher events.^48^

Furthermore, selectivity of the redirected effector cell population may improve tolerability (pan-T cell populations versus γδ T-cell-specific TCEs).^49^ CD3 engagement lacks recruitment selectivity, given the interaction with both CD8+ and CD4+ naïve T-cells. Polyclonal T-cell activation of regulatory T-cells and other CD4+ subpopulations like TH_1_ and TH_17_ may result in an overall counterproductive T-cell suppression with limited tumor cell killing and rapid secretion of cytokines leading to CRS.^50^ Next-generation antibodies have been developed in order to improve selective recruitment of CD8+ cytotoxic T lymphocytes over pan-CD3+ T-cell redirection. Similarly, non-conventional T-cell selective activation can exploit specific properties of T-cell subgroups. γδ T Cell Bispecific Antibody Adapters (Gammabody®), for example, can selectively recruit γδ T-cells, which activate their immune effector response independently of the antigen presenting machinery and, importantly, release lower levels of pro-inflammatory cytokines compared to the classical αβT-cell receptor cells.^51^

Therapeutic strategies to mitigate CRS include IL-6 pathway blockade with tocilizumab or IL-6-targeting agents such as siltuximab or clazakizumab, which do not appear to impair antitumor responses, as well as corticosteroids. Initial clinical evidence suggests that preemptive cytokine modulation with tocilizumab or corticosteroids may attenuate early inflammatory cascades and mitigate immune-mediated toxicities. Additionally, kinase inhibitors, including JAK and mTOR inhibitors, may contribute to reducing cytokine production while preserving T-cell cytotoxicity, though their clinical role remains to be fully elucidated.^52^^,^^53^

Immune effector cell–associated neurotoxicity syndrome (ICANS) is a neurologic adverse event, often co-occurring with CRS, in which cytokine-mediated endothelial dysfunction results in disruption of the blood–brain barrier and transient central cytokine leak. Although CRS and ICANS share upstream T-cell activation and endothelial injury, CRS predominantly reflects IL-6–driven peripheral vascular dysfunction, while ICANS is more tightly linked to IL-1–mediated neuroinflammation, blood–brain barrier (BBB) breakdown, and disturbance of neuronal excitability.^52^^,^^54^ Early symptoms include tremor, dysgraphia, aphasia, apraxia, and lethargy, with potential progression to seizures, encephalopathy, or coma. The Immune Effector Cell–Associated Encephalopathy (ICE) score is routinely used for objective clinical assessment of ICANS. Tocilizumab does not cross the blood–brain barrier and is ineffective in this setting; therefore, corticosteroids remain the mainstay of management. The incidence of ICANS with bsAbs is generally low (typically <5%) but varies by agent and tumor type. In solid tumors, a higher incidence of ICANS has been reported with bsAbs TCE targeting DLL3 (tarlatamab, 8–28%; HPN328, 9%), partially attributed to on-target/off-tumor effects on neural tissues.^55^

On-target/off-tumor toxicity occurs when the bsAb binds to its intended antigen, but antigen expression is shared with normal tissues. Multiple examples have been reported, sometimes limiting treatment continuation, such as gastrointestinal toxicities observed with cibisatamab (CEA x CD3 bsAb) and zanidatamab or skin toxicity with EGFR x MET bsAbs.^29^^,^^30^ Specificity, selectivity and valency contribute to the risk of these adverse events. High specificity defines the capacity of the drug to bind exclusively to the target without cross-reacting, whereas selectivity is influenced by tumor heterogeneity and antigen density in normal tissues, that may lead to unintended binding. Asymmetric formats, as the 2 + 1 trivalent bsAb with two binding sites for the TAA and one for an immune effector cell antigen enhance preferential binding to tumor cells with high antigen density.^56^

Infusion-related reactions (IRRs) typically present within minutes to hours of drug administration and range from mild fever and rash to severe anaphylaxis; in the case of bsAb, IRRs are common during the first infusion, as exemplified by amivantamab and tarlatamab, requiring prophylactic and management strategies.^57^ For subcutaneously administered bsAbs, while IRRs are less frequent, injection site reactions (ISRs) are commonly reported. ISRs include localized erythema, swelling, pruritus, or pain at the site of administration and are frequently mild and self-limiting. However, ISRs may require symptomatic treatment and should be anticipated.^58^

Anti-drug antibodies (ADAs) are host-derived immune responses generated against therapeutic antibodies. Although ADAs are most often non-neutralizing, they may alter pharmacokinetics (PK), reduce drug efficacy, and in some cases, induce hypersensitivity. This was observed with cibisatamab, where ADA formation was associated with significant loss of drug exposure, compromising therapeutic efficacy. Mitigation strategies include premedication with corticosteroids and antihistamines, controlled infusion rates, and route adjustments (e.g., IV over SC). More advanced approaches include B-cell depletion using anti-CD20 agents or emerging techniques such as deimmunization via protein engineering to minimize immune recognition, but these are not broadly used yet.^59^

The immunopharmacology of the Fc region can be modulated to the desired pharmacological activity. Fc silencing through the introduction of mutations can attenuate the unwanted interaction with Fc gamma receptors (FcγR) and thus with immune cells, limiting inflammatory and auto-immune events, as well as cytokine release. In spite of the intended Fc silencing, binding of neonatal Fc receptor (FcRn) maintains retained, an important feature to regulate the half-life of IgG antibodies by recycling them back into circulation. L234A/L235A (“LALA”) is the most commonly used IgG1 variant, which leads to absence of complement-dependent cytotoxicity and 100-fold lower binding of FcγRI and FcγRII. However, the silencing is incomplete: the residual binding to soluble FcγRI and to 158 V allele of FcγRIIIa maintain high levels of ADCP and ADCC.^60^ Other mutations can be incorporated, as explored with cadonilimab to achieve a nearly completely silenced Fc domain; however, thermal stability and proteolytic degradation can be compromised. The “STR” variant (L234S/L235T/G236R) has been adopted by the World Health Organization as the gold standard for silencing.^61^ Opposite to silencing, Fc fucosylation, sialylation and glycosylation regulate the intensity of the inflammatory response, recruitment of inflammatory cells and regulation of pro-inflammatory cytokines release as well as antitumor immunity via FcγR signaling and cellular effector functions.^62^

### Special dosing considerations

The efficacy and safety of bsAbs are driven not only by molecular design, but also by dosing strategy and route of administration. Given their complexity, conventional fixed dosing can lead to excessive toxicity or suboptimal immune activation. Adapted approaches, including step-up and split dosing, or subcutaneous (SC) delivery, are increasingly used to prevent such effects.

During the first treatment cycle, step-up dosing involves a sequence of escalating priming doses designed to gradually reach the full therapeutic dose. This approach promotes immune desensitization and mitigates CRS. Its utility is well supported by PK and pharmacodynamic (PK/PD) modeling as well as exposure–CRS analyses across several FDA-approved bsAbs, including tebentafusp and various T-cell engagers used in hematologic malignancies.^63^

Another approach employed during the same initial phase is the use of split dosing, in which the full therapeutic dose is divided into smaller fractions administered sequentially over a defined period, typically within the first cycle, to modulate PD and improve tolerability, in particular for highly potent bsAbs. This was the case with amivantamab and zenocutuzumab, both of which demonstrated that split dosing is crucial to reduce the incidence and severity of IRRs, likely due to Fcγ receptor–mediated effects and target expression in normal tissues.^24^^,^^52^^,^^64^

**Fig. 3:**
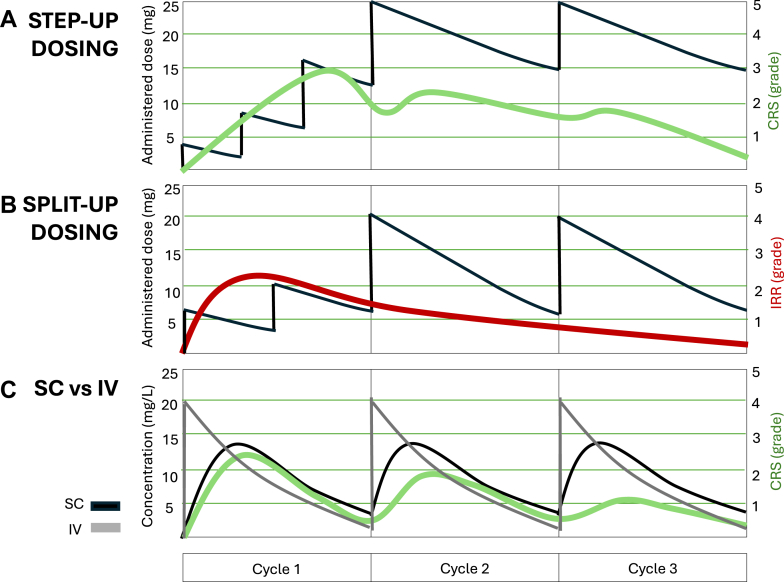
**Conceptual illustration of dose optimization strategies and their potential impact on toxicity prevention.** A. Step-up dosing: During the first cycle, gradually increasing doses are administered to enable progressive immune priming. B. Split-up dosing: The total dose is divided into two or more equal parts during the first cycle to reduce the risk of severe infusion-related reactions (IRRs). C. Subcutaneous (SC) versus intravenous (IV) administration: Lower Cmax with similar exposure and delayed Tmax following SC administration results in reduced acute immune activation and a lower risk of cytokine release syndrome (CRS).

Subcutaneous (SC) administration is increasingly preferred due to reduced clinic time and improved patient comfort, but it also offers PK advantages that may impact in better outcomes. Compared to intravenous (IV) delivery, SC administration generally yields a lower Cmax, delayed Tmax, and higher Cmin, which may lead to more stable drug exposure and extended dosing intervals (Fig. 3). For instance, SC amivantamab resulted in a marked reduction in IRRs (67% IV vs. 16% SC) without compromising exposure. Similarly, SC mosunetuzumab was well tolerated with no severe CRS in B-cell non-Hodgkin lymphoma patients, likely due to a lower peak-to-trough ratio facilitating gradual immune priming. However, SC administration may pose a higher risk for ADA formation due to antigen trafficking through local lymphatic vessels. Despite the fact that ADAs do not necessarily have clinical relevance, for JNJ-63898081, a CD3 x PSMA bsAb, SC administration led to higher levels of neutralizing ADAs and study discontinuation. Nevertheless, further evaluation is needed, as ADA formation has been observed at variable frequencies, particularly in bsAbs that do not deplete B cells (e.g., 15–35% for talquetamab), but reported titers are generally low, and current data do not suggest a meaningful impact on pharmacokinetics, safety, or clinical efficacy.^27^^,^^64^^,^^65^

### Mechanisms of resistance

#### Tumor-intrinsic: antigen modulation and an immunosuppressive microenvironment

While antigen modulation is well-documented in hematologic malignancies, emerging evidence indicates similar challenges in solid tumors.^66^ Mechanisms such as EpCAM downregulation and HER2 extracellular domain shedding have shown to limit the efficacy of bsAbs like solitomab and zanidatamab by enabling immune escape and reducing target engagement. Developing therapies that target multiple antigens simultaneously may reduce the likelihood of resistance due to the loss of a single antigen.^34^^,^^67^

Antigen presentation can be affected by MHC-I downregulation as well. Although bsAbs can function independently of MHC-TCR recognition, MHC-I loss still hinders effective T-cell activation, particularly in the priming phase, by disrupting antigen presentation and subsequent recruitment and activation of naïve CD8^+^ T cells.^68^

The immunosuppressive TME is a major barrier to bsAb efficacy in solid tumors by impairing T-cell infiltration, activation, and function. The accumulation of regulatory T cells (Tregs), myeloid-derived suppressor cells (MDSCs), and inhibitory cytokines such as TGF-β and IL-10 leads to local immune suppression, which undermines the immune-mediated cytotoxicity initiated by BsAbs.^56^

#### Immune-related: T-cell exhaustion

T-cell dysfunction, marked by reduced proliferation, impaired cytokine production, and diminished cytotoxicity capacity, can be exacerbated by chronic stimulation from bsAbs, contributing to exhaustion and treatment failure. Targeting inhibitory or co-stimulatory pathways may offer a strategy to reverse exhaustion.

#### Drug-related: clearance

The sink effect refers to the phenomenon in which a therapeutic antibody is unexpectedly and rapidly cleared from circulation, resulting in reduced half-life and drug exposure. Traditionally, this has been attributed to binding of the antibody to its target antigen in normal tissues. Furthermore, a conceptually similar mechanism occurs when antibodies undergo Fc-mediated degradation, a process in which the Fc region engages activating FcγRs on immune cells, leading primarily to ADCP and ADCC.^69^^,^^70^ Fc-mediated degradation can be minimized through Fc-silencing with FcRN binding preserved to prevent uptake by immune cells, while tumor-selective drug designs, such as pH-dependent binding, enhance specificity. Pharmacologically, high loading doses and frequent administration help saturate peripheral sinks and maintain therapeutic levels.^71^

While non-IgG-like bsAbs are more often antibody fragments with small dimensions for improved tumor permeability and tissue distribution, in addition to a lower risk of non-specific activation of innate immunity,^72^ a significant drawback is the intrinsic fast clearance with short half-life and consequently limited drug exposure. The incorporation of a human serum albumin (HSA)-binding domain in the antibody scaffolding structure is a well-established strategy to increase half-life,^73^ while multimerization with peptide linkers and PEGylation are also being explored for the same purpose.^66^ Conversely, a Self-Assembling and Disassembling (SADA) BsAb platform is in development to deliver high doses of a bsAb that self-assemble into a stable tetramer with antitumor activity but can fast disassemble into dimers or monomers that can rapidly be excreted via the kidney, also reducing immunogenicity.^74^ Furthermore, the incorporation of an Fc domain within antibody fragments resulting in longer half-life next-generation molecules is currently under clinical investigation, such as with HLE-BiTEs® (NCT05740566).

## Future perspectives

The development of bispecific and multi-specific antibodies is rapidly evolving from hematological malignancies to solid tumors, although not without challenges mostly related to drug activity, tissue penetrance and safety. Next generation-antibody structures aim to address these challenges in order to increase anticancer activity via multi-valency or the addition of payloads, or increase the drug targeted delivery to cancer and tumor-microenvironment while sparing normal cells.

Compared to other anti-cancer strategies in current development, bsAbs represent an innovative approach with different mechanism of action compared to chemotherapy, which still remains the only standard approach available for the vast majority of cancers. Their delivery is feasible in an outpatient facility with some additional requirements for monitoring of CRS symptoms in case of cell-bridging agents. This is increasingly performed via wearable devices and telemedicine, reducing hospitalization and costs, making bsAbs a scalable and attractive option compared to more complex therapies in development as example cellular therapies. Similarly to monoclonal antibodies, most agents have a safety profile suitable for combination therapies, in particular with establishes immune-checkpoint inhibitors or standard chemotherapy, which is a favorable characteristic compared to antibody drug conjugates. Limited cumulative toxicity compared to chemotherapy-based agents, make bsAbs suitable for long-term maintenance strategies. However, limited single agent activity especially in immunogenically cold tumors requires further improvements.

### Next-generation antibody design

#### Innovative antibody structures

##### Multivalency

Multivalency antibodies have an increased number of Fab regions, leading to enhanced activity via simultaneous mechanisms of action within the same molecule. Trispecific TCEs (TriTCEs), which integrate a co-stimulatory receptor like CD28, ICOS, OX40 or 4-1BB, provide a second signal to the TCR aiming to increase activity in poorly infiltrated tumours.^75^^,^^76^ Furthermore, GNC-039, a tetraspecific antibody simultaneously targeting EGFRvIII x CD3 x PD-L1 x 4-1BB can function as both cell-engager by bridging CD3 and EGFRvIII while increasing T-cell activation via dual checkpoint inhibition of PD-L1 and 4-1BB.^77^

##### Adjunctive component

Following the evolution of monoclonal antibodies into antibody-drug conjugates (ADCs), the logical next step for bsAbs was the addition of a payload. This is expected to increase cell killing via the payload's mechanism of action, increase the anti-tumor activity via by-stander effect and overcome expression heterogeneity of the target antigens. A number of bsAb-drug conjugates are currently in clinical development, including BL-B01D1 (EGFR x HER3) and AZD9592 (EGFR x c-MET), both carrying a topoisomerase I inhibitor payload, and exhibiting preliminary antitumor activity and acceptable safety profile^78^; zanidatamab-zovodotin (biparatopic HER2 bsAb, carrying a novel auristatin payload), YH012 (HER2 x TROP2; MMAE payload), and M1231 (MUC1 x EGFR; a hemiasterlin-related microtubule inhibitor payload).

Non-cell bridging bsAbs are particularly suitable for cytotoxic payloads; conversely, cell-engager activity can be further enhanced by the incorporation of an immunomodulatory payload like a cytokine (mostly Il-2, IL12 or IL-15), toll-like receptor 7/8 agonists or a small interfering RNA (siRNA) payloads.^79^

Moreover, novel bsAbs constructs are being developed by combining the antitumoral properties of PROteolysis-TArgeting Chimeras (PROTACs), and the dual activity of bsAb arms: EpiTACs are modular bsAbs including one arm targeting a cancer cell surface target and a degrader binding arm, targeting a transmembrane E3 ubiquitin ligase degrader.^80^

##### Refined activation

Masking technologies with unmasking of the Fab only in presence of low pH or matrix metalloproteinases present in the tumor microenvironment (TME) mitigate the risk of on-target/off-tumor drug activation.^81^ These are applicable equally to monoclonal antibodies and bsAbs, both non-cell bridging as well as cell-engagers, such as the COBRA™ (COnditional Bispecific Redirected Activation) platform, administered as pro-drugs that remain inert in healthy tissues and in circulation, while acquiring an active TCE conformation only within the TME (NCT04844073, NCT05220098).^82^ Similarly, the Prodrug-Activating chain exchange (PACE) technology allows for the infusion of two separate pro-drug chains that are activated upon close proximity: one prodrug accumulates on the cell surface via the TAA binding domain; the second prodrug converts the two chains into a TCE active conformation of a bi- or tri-specific antibody directly on the cancer cell, overcoming the need to rely on specific TME conditions.^83^

Another promising strategy is to leverage the mRNA vaccine technology to synthesize anti-cancer bsAbs in vivo. RiboMabs (bispecific monoclonal antibody-encoding mRNA) are lipid nanoparticle-encapsulated RNAs that encode for a T-cell-engaging bispecific antibody, with the advantage of endogenous hepatic production and, therefore, minimized undesired adverse immune reactions. Anti-tumor activity has been broadly shown in animal models,^84^ and two compounds are under clinical assessment, such as BNT-142 (RiboMab02.1), a CD3 x CLDN6 in patients with prospectively confirmed claudin 6-positive solid tumors.^85^

### Combination therapies

Combining a bsAb with an additional anticancer drug can be explored under different rationales. From a safety standpoint, a second agent can be deployed to reduce the tumor burden with consequent reduction of the antigen compartment and subsequent mitigation of CRS risk. Such depletion strategy is particularly effective in hematological malignancies, with expected fast responses and competing target antigen receptor occupancy via combination with an anti-CD20 monoclonal antibody, chemotherapy or CPIs.^86^

In contrast, by prioritizing efficacy, the combining agent can be used to increase the expression of the target antigen, as observed with CD20 expression in large B cell lymphoma upon treatment with the CD79b-targeting ADC polatuzumab vedotin (Pola), making the Pola-refractory cells more sensitive to the TCE mosumetuzumab.^87^

Additionally, synergistic activity can also be achieved. Fragments of intracellular oncoproteins—such as mutant KRAS or EGFR—can act as neoantigens presented by MHC upon treatment with their corresponding covalent-inhibitors, which may then be targeted by “HapImmune” TCE antibodies.^88^ Furthermore, a special class of TCEs has been developed to act as an adaptor between CAR-T and cancer cells: by recognizing a TAA on cancer surface and the synthetic CAR antigen receptor, there is no engagement of natural T-cells, moving beyond the standard mechanism of action for both bsAbs and cellular therapy.^89^

Panel 1 **Key implications of bispecific and multispecific antibodies in oncology.**

This panel summarises key concepts in the main text and corresponding references therein

1. Classifications: structural and mechanistic frameworks.
  - Use structural classification (IgG-like versus non-IgG-like formats, valency, Fc presence and configuration) to align each construct with its intended pharmacokinetic profile, tissue penetration and need for Fc-mediated effector functions or Fc silencing in a given indication.
  - Apply mechanistic classification (cell-engaging bsAbs/msAbs, non–cell-bridging immune modulators, and receptor–ligand blockade) to match antibody design to disease biology.
  - For multispecific antibodies, define a clear and testable biological rationale for each additional specificity (second tumour antigen, costimulatory receptor, or microenvironmental target), explicitly linked to known patterns of tumour heterogeneity, immune escape, or stromal remodelling.
2. Recent achievements in clinical development.
  - EMA has approved multiple T-cell–engaging bsAbs in B-cell neoplasms, including the CD19 × CD3 BiTE blinatumomab for B-cell precursor ALL; the BCMA × CD3 bsAbs teclistamab, elranatamab, and linvoseltamab and the GPRC5D × CD3 bsAb talquetamab for relapsed/refractory multiple myeloma; and the CD20 × CD3 bsAbs mosunetuzumab (follicular lymphoma), glofitamab, and epcoritamab (diffuse large B-cell lymphoma), illustrating the maturity of this modality across B-cell malignancies.
  - In solid tumours, the CD3 × HLA-A∗02:01-gp100 ImmTAC tebentafusp has, for the first time, demonstrated a clinically meaningful overall-survival benefit in metastatic uveal melanoma, while amivantamab (EGFR × MET) has become the first FDA and EMA-approved bsAb for a solid malignancy, with phase 3 data consolidating its role across multiple EGFR-mutant NSCLC settings.
3. Challenges and considerations.
  - Anticipate and manage the characteristic toxicity spectrum of bsAbs (CRS, ICANS, on-target/off-tumour effects, infusion-related reactions, and anti-drug antibodies) through standardized grading, early intervention algorithms and target-specific mitigation strategies.
  - Integrate dose and schedule as deliberate design variables: use step-up or split dosing and, where appropriate, subcutaneous administration to modulate peak concentrations and cytokine surges, while maintaining sufficient exposure for sustained target engagement.
  - Address mechanisms of resistance at three levels: tumour-intrinsic (antigen modulation, antigen presentation defects, immunosuppressive microenvironment), immune-related (T-cell exhaustion) and drug-related (sink effect, rapid clearance) when selecting targets, formats, and combination partners, and when planning biomarker-driven correlative studies.
4. Future perspectives.
  - Prioritise next-generation multivalent and multispecific architectures that exploit increased avidity, dual checkpoint modulation, or concurrent engagement of tumour and immune targets, if they demonstrably improve functional potency or selectivity over simpler constructs.
  - Develop bsAbs and msAbs with adjunctive components (such as cytotoxic payloads, cytokine moieties or other functional modules) and with refined, context-dependent activation to concentrate activity within the tumour and its microenvironment and limit systemic toxicity.
  - Systematically explore rational combinations of bsAbs/msAbs with other anticancer agents (chemotherapy, targeted therapies, immune checkpoint inhibitors, and cellular therapies), guided by mechanistic complementarity and resistance patterns, and supported by prospective translational programmes that monitor antigen expression, immune-cell dynamics and microenvironmental changes over time.

## Conclusions

We have summarized the complexity of constructs, clinical successes, challenges in administration and resistance mechanisms in the fast-growing world of multi-specific antibody-based therapy. Although these agents are moving precision medicine forward, the success rate from drug discovery to clinical adoption remains low. Preclinical models have limitations, in particular when attempting to recreate tumor microenvironment conditions required for drug activation, cellular interactions with different immune components and tumor heterogeneity. Similarly, dynamic changes induced upon treatment are difficult to predict, hampering the design of rational synergistic combinations.

Although selected based on expected ubiquitous expression according to tumor type, TAA heterogeneity and antigen loss are gradually driving us towards biomarker testing for patient selection. This is often via immunohistochemistry which remains operator-dependent and difficult to standardize. Progress with digital pathology will hopefully facilitate selection criteria. At present, for most approved bispecific antibodies, biomarker-driven selection largely relies on confirming target expression or key oncogenic drivers, while more complex predictive signatures remain exploratory and confined to translational substudies.

Often these agents enter clinical trials in heavily pre-treated populations: prior exposure to drugs with the same target or the limited activity as monotherapy may have contributed to early termination of several programs. The radical change in the design of early-phase clinical trials, including multi-cohort platforms for dose optimization and combinatorial strategies, biomarker selection and inclusion of treatment-naïve patients has been essential to enable a thorough investigation of their full potential, bringing at least 13 agents so far to standard of care and hopefully many more in the near future.

## Contributors

E.F.: conceptualization, writing original draft, supervision, writing-review and editing.

R.G.: conceptualization, writing original draft, supervision, writing-review and editing.

N.K.: conceptualization, writing original draft, writing-review and editing.

B.D.: conceptualization, writing original draft, writing-review and editing.

S.N.: conceptualization, writing original draft, writing-review and editing.

M.M.: conceptualization, writing original draft, supervision, writing-review and editing.

## Declaration of interests

Elisa Fontana: Support for attending meetings and/or travel from Repare Therapeutics, CARIS Life Science, Seagen, Sapience, BicycleTx Ltd, Erasca; Participation on a Data Safety Monitoring Board or Advisory Board from Astellas, Pfizer, BicycleTx Ltd, BMS, Erasca; leadership from European Organisation for Research and Treatment of Cancer (EORTC), GITCG secretary (2021–2023) ASCO Annual Meeting Scientific Programme Committee GI cancers, Colorectal and Anal Track (2024–2026); Other financial or non-financial interests from Acerta Pharma, ADC Therapeutics, Amgen, Arcus Biosciences, Array BioPharma, Artios Pharma Ltd, Astellas Pharma Inc, Astex, Astra Zeneca, Basilea, Bayer, BeiGene, BicycleTx Ltd, BioNTech, Blueprint Medicines, Boehringer Ingelheim, Calithera Biosciences, Inc., Carrick Therapeutics, Casi Pharmaceuticals, Clovis Oncology, Inc, Crescendo Biologics Ltd., CytomX Therapeutics, Daiichi Sankyo, Deciphera, Eli Lilly, Ellipses, Erasca, Exelixis, F. Hoffmann-La Roche Ltd, Fore Biotherapeutics, G1 Therapeutics, Genentech, GSK, H3 Biomedicine Inc, Hutchinson MediPharma, Ignyta/Roche, Immunocore, Immunomedics, Inc., Incyte, Instil Bio, IOVANCE, Janssen, Jiangsu Hengrui, Kronos Bio, Lupin Limited, MacroGenics, Menarini, Merck KGaA, Mereo BioPharma, Merus, Millennium Pharmaceuticals, MSD, Nerviano Medical Sciences, Nurix Therapeautics Inc, Oncologie, Oxford Vacmedix, Pfizer, Plexxikon Inc., QED Therapeutics, Inc., Relay Therapeutics, Repare Therapeutics, Ribon Therapeutics, Roche, Sapience, Seagen, Servier, Stemline, Synthon Biopharmaceuticals, Taiho, Tesaro, Turning Point Therapeutics, Inc, PMVPharma, Takeda.

Rafael Grochot: none.

Nuria Kotecki: Consulting fees from Daiichi; Payment or honoraria for lectures, presentations, speakers bureaus, manuscript writing or educational events from AZ and Gilead; Support for attending meetings and/or travel from AZ and Byondis; Leadership at Medical Executive officer at Oncodistinct network.

Bernard Doger: none.

Simon Nannini: none.

María de Miguel: Consulting fees from Pharmamar; Payment or honoraria for lectures, presentations, speakers bureaus, manuscript writing or educational events from HiFiBio; Support for attending meetings and/or travel from Lilly; Participation on a Data Safety Monitoring Board or Advisory Board from MSD and J&J.

## Appendix B Supplementary data

Summary French

Summary Spanish
