# Supplementary material for: Evolution of bispecific and multispecific antibodies in cancer therapy
Source: Lancet Reg Health Eur. 2026 Mar 19;64:101599. doi: 10.1016/j.lanepe.2026.101599 (PMC13147840; doi:10.1016/j.lanepe.2026.101599)
Supplement: Summary French [file mmc1.docx]

Ref.: TLRHEUROPE-D-25-00583

**SUMMARY-FRENCH**

La thérapie anticancéreuse fondée sur les anticorps a évolué rapidement, passant des anticorps monoclonaux à des constructions bispécifiques et multispécifiques combinant des spécificités de liaison et des mécanismes d’action distincts. Ces agents connaissent une adoption clinique croissante, comme en témoignent les autorisations de l’Agence européenne des médicaments dans les hémopathies malignes et dans certains cancers solides, notamment le mélanome uvéal et le cancer du poumon non à petites cellules avec mutation d’EGFR. Pourtant, ils sont encore souvent présentés comme une seule classe thérapeutique, ce qui ne reflète pas la complexité des formats et des mécanismes actuels, allant d’architectures de type IgG à des formats à base de fragments, et de la redirection des cellules immunitaires à la modulation immunitaire double ou au blocage de voies de signalisation oncogéniques.

Cet article de la Série propose un cadre de classification intégré fondé sur le format et le mécanisme d’action, en reliant des caractéristiques clés de conception à la pharmacologie, à l’efficacité et à la sécurité. Les données cliniques et le développement en cours sont synthétisés, des stratégies pratiques visant à atténuer les toxicités caractéristiques sont discutées, et les mécanismes émergents de résistance ainsi que des approches rationnelles d’associations thérapeutiques sont examinés. Sont également présentées les orientations de prochaine génération, incluant des constructions multispécifiques d’ordre supérieur, des anticorps à activité conditionnelle, et des formats multispécifiques conjugués à une charge.

Afin de consolider ces agents comme une modalité thérapeutique établie en oncologie, il convient de privilégier une compréhension rigoureuse des mécanismes d’action et de la toxicité, ainsi qu’une optimisation rationnelle de la conception des constructions et de la posologie, appuyée par des programmes translationnels prospectifs robustes.
