# Supplementary material for: Evolution of bispecific and multispecific antibodies in cancer therapy
Source: Lancet Reg Health Eur. 2026 Mar 19;64:101599. doi: 10.1016/j.lanepe.2026.101599 (PMC13147840; doi:10.1016/j.lanepe.2026.101599)
Supplement: Summary Spanish [file mmc2.docx]

Ref.: TLRHEUROPE-D-25-00583

**SUMMARY-SPANISH**

La terapia oncológica basada en anticuerpos ha evolucionado rápidamente desde los anticuerpos monoclonales hasta constructos biespecíficos y multiespecíficos, que combinan especificidades de unión y mecanismos de acción distintos. Estos agentes están experimentando una adopción clínica creciente, con aprobaciones de la Agencia Europea de Medicamentos en neoplasias hematológicas y en tumores sólidos seleccionados, como el melanoma uveal y el cáncer de pulmón no microcítico con mutación de EGFR. Sin embargo, a menudo siguen considerándose como una única clase farmacológica, lo que no refleja la complejidad de los formatos y mecanismos actuales, que abarcan desde arquitecturas tipo IgG hasta formatos basados en fragmentos, y desde la redirección de células inmunes hasta la modulación inmunitaria dual o el bloqueo de vías oncogénicas.

Este artículo se proporciona un marco de clasificación integrado basado en mecanismos y formatos, relacionando características clave del diseño con la farmacología, la eficacia y la seguridad. Sintetiza la evidencia clínica y el desarrollo en curso, analiza estrategias prácticas para mitigar toxicidades características y revisa mecanismos emergentes de resistencia y enfoques racionales de combinación. También se desarrollan las nuevas generaciones de estos fármacos, incluidos constructos multiespecíficos de orden superior, anticuerpos con activación condicionada y formatos multiespecíficos conjugados con carga.

Para consolidar estos agentes como una modalidad terapéutica establecida en oncología, debe priorizarse una comprensión rigurosa de los mecanismos de acción y de la toxicidad, junto con la optimización racional del diseño del constructo y de la administración, respaldada por programas traslacionales prospectivos y sólidos.
